# *N*,*N*′-Bis(4-bromo­benzyl­idene)butane-1,4-diamine

**DOI:** 10.1107/S1600536808028122

**Published:** 2008-09-06

**Authors:** Hoong-Kun Fun, Hadi Kargar, Reza Kia

**Affiliations:** a X-ray Crystallography Unit, School of Physics, Universiti Sains Malaysia, 11800 USM, Penang, Malaysia; b Department of Chemistry, School of Science, Payame Noor University (PNU), Ardakan, Yazd, Iran

## Abstract

The mol­ecule of the title Schiff base compound, C_18_H_18_Br_2_N_2_, lies across a crystallographic inversion centre and adopts an *E* configuration with respect to the C=N bond. In the crystal structure, mol­ecules are linked into chains along [201] through inter­molecular Br⋯Br inter­actions [3.3747 (3) Å], which are significantly shorter than the sum of the van der Waals radii for Br atoms (3.70 Å). The crystal structure is further stabilized by π–π stacking inter­actions [centroid–centroid distance 3.6811 (11) Å].

## Related literature

For halogen–halogen inter­actions, see: Ramasubbu *et al.* (1986 ▶); Brammer *et al.* (2003 ▶). For the crystal structures of related compounds, see: Fun *et al.* (2008 ▶); Fun, Kia & Kargar (2008*a* ▶,*b* ▶); Fun & Kia (2008*a* ▶,*b* ▶). For bond-length data, see: Allen *et al.* (1987 ▶). For hydrogen-bondong motifs, see: Bernstein *et al.* (1995 ▶). For background, see: Casellato & Vigato (1977 ▶).
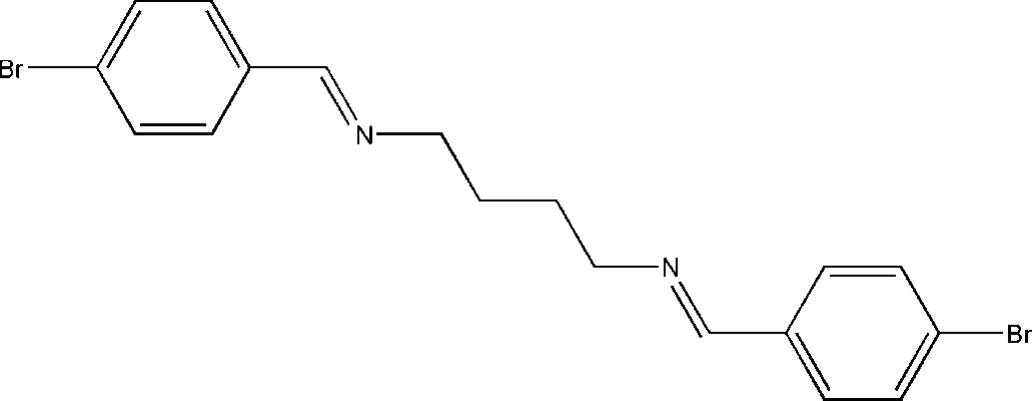


## Experimental

### 

#### Crystal data

C_18_H_18_Br_2_N_2_
*M* *_r_* = 422.16
Monoclinic, 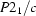

*a* = 11.2612 (5) Å
*b* = 9.5213 (4) Å
*c* = 8.2645 (4) Å
β = 100.040 (3)°
*V* = 872.56 (7) Å^3^
*Z* = 2
Mo *K*α radiation
μ = 4.64 mm^−1^
*T* = 100.0 (1) K
0.52 × 0.23 × 0.08 mm

#### Data collection

Bruker APEXII CCD area-detector diffractometer
Absorption correction: multi-scan (*SADABS*; Bruker, 2005 ▶) *T*_min_ = 0.192, *T*_max_ = 0.688
15460 measured reflections
3843 independent reflections
2600 reflections with *I* > 2σ(*I*)
*R*_int_ = 0.037

#### Refinement

*R*[*F* ^2^ > 2σ(*F* ^2^)] = 0.037
*wR*(*F* ^2^) = 0.092
*S* = 1.02
3843 reflections
136 parameters
All H-atom parameters refined
Δρ_max_ = 0.68 e Å^−3^
Δρ_min_ = −0.59 e Å^−3^

### 

Data collection: *APEX2* (Bruker, 2005 ▶); cell refinement: *SAINT* (Bruker, 2005 ▶); data reduction: *SAINT*; program(s) used to solve structure: *SHELXTL* (Sheldrick, 2008 ▶); program(s) used to refine structure: *SHELXTL*; molecular graphics: *SHELXTL*; software used to prepare material for publication: *SHELXTL* and *PLATON* (Spek, 2003 ▶).

## Supplementary Material

Crystal structure: contains datablocks global, I. DOI: 10.1107/S1600536808028122/rz2242sup1.cif

Structure factors: contains datablocks I. DOI: 10.1107/S1600536808028122/rz2242Isup2.hkl

Additional supplementary materials:  crystallographic information; 3D view; checkCIF report

## Figures and Tables

**Fig. 1.**
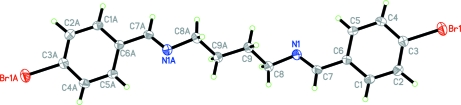
The molecular structure of the title compound with atom labels and 50% probability ellipsoids for non-H atoms. The suffix A corresponds to symmetry code (-x, -y + 1, -z).

**Fig. 2.**
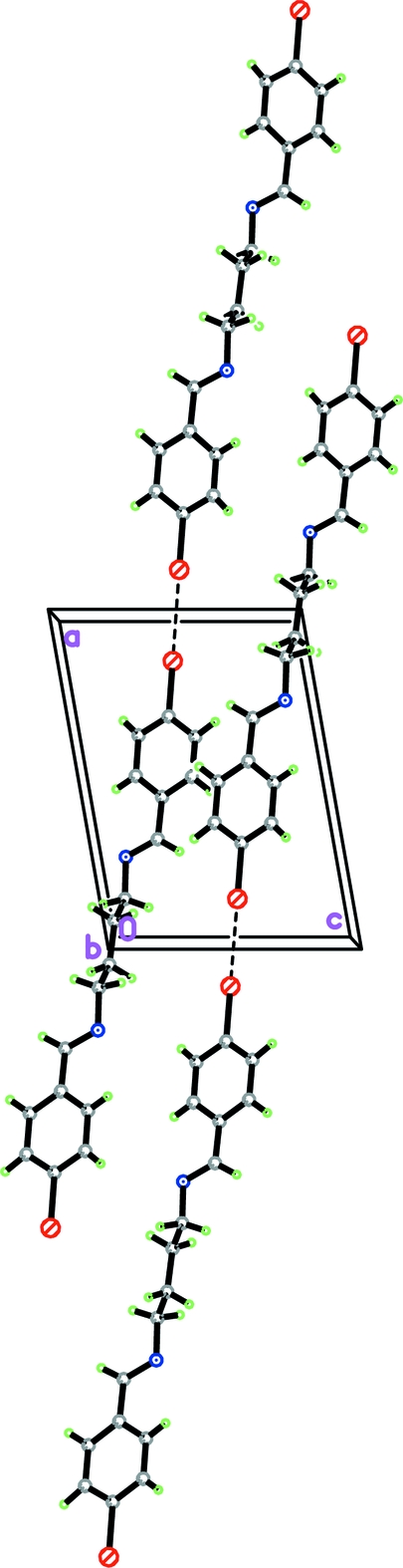
The crystal packing of the title compound viewed down the b axis, showing molecules linked into chains along the [201] direction by short intermolecular Br···Br interactions.

**Fig. 3.**
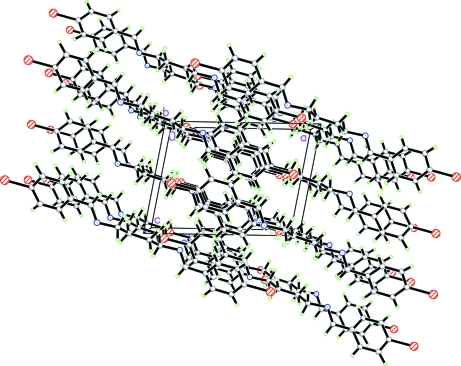
The crystal packing of the title compound viewed down the b-axis, showing the π–π stacking arrangement of molecules.

## supplementary crystallographic information

### Comment

The condensation of primary amines with carbonyl compounds yields Schiff bases (Casellato & Vigato, 1977) that are still one of the most prevalent mixed-donor ligand in coordination chemistry. In the past two decades, the synthesis, structure and properties of Schiff base complexes have stimulated much interest for their noteworthy contributions in single molecule-based magnetism, materials science, catalysis of many reactions like carbonylation, hydroformylation, reduction, oxidation, epoxidation and hydrolysis (Casellato & Vigato 1977). As an extension of our work (Fun *et al.*,  2008; Fun, Kia & Kargar 2008*a*,*b*; Fun & Kia 2008*a*,*b*) on the structural characterization of Schiff base ligands, the title compound is reported here.

The molecule of the title compound (Fig 1), lies across a crystallographic inversion centre and adopts an *E* configuration with respect to the C═N bond. The bond lengths (Allen *et al.*, 1987) and angles are within normal ranges. The asymmetric unit of the compound is composed of one-half of the molecule. The imino group is coplanar with the benzene ring. Within the molecule, the planar units are parallel but extend in opposite directions from the methylene bridge. An interesting feature of the crystal structure is the short Br···Br [3.3747 (3) Å] interaction (Fig. 2), which is significantly shorter than the sum of the van der Waals radii for two Br atoms (3.70 Å). The directionality of these interactions, C—X···X—C (X = halogens), has been attributed to anisotropic van der Waals radii for terminally bound halogens or ascribed to donor–acceptor interactions involving a lone pair donor orbital on one halogen and a C—X σ^*^ acceptor orbital on the other (Ramasubbu *et al.*, 1986; Brammer *et al.*, 2003). In the crystal structure, molecules are linked into chains along the [201] direction through the short intermolecular Br···Br interactions (Fig. 2). In addition, the crystal structure is further stabilized by π–π interaction (Fig. 3) with centroid-to-centroid distance of 3.6811 (11) Å, perpendicular interplanar distance of 3.3617 (8) Å, and centroid···centroid offset of 1.4997 (5) Å.

### Experimental

The synthetic method has been described earlier (Fun, Kia & Kargar, 2008*b*). Single crystals suitable for X-ray diffraction were obtained by evaporation of an ethanol solution at room temperature.

### Refinement

All hydrogen atoms were located from the difference Fourier map and refined freely.

### Crystal data

**Table:**

C_18_H_18_Br_2_N_2_	*F*(000) = 420
*M**_r_* = 422.16	*D*_x_ = 1.607 Mg m^−^^3^
Monoclinic, *P*2_1_/*c*	Mo *K*α radiation, λ = 0.71073 Å
Hall symbol: -P 2ybc	Cell parameters from 5047 reflections
*a* = 11.2612 (5) Å	θ = 2.8–33.0°
*b* = 9.5213 (4) Å	µ = 4.64 mm^−^^1^
*c* = 8.2645 (4) Å	*T* = 100 K
β = 100.040 (3)°	Plate, colourless
*V* = 872.56 (7) Å^3^	0.52 × 0.23 × 0.08 mm
*Z* = 2	

### Data collection

**Table:**

Bruker SMART APEXII CCD area-detector diffractometer	3843 independent reflections
Radiation source: fine-focus sealed tube	2600 reflections with *I* > 2σ(*I*)
graphite	*R*_int_ = 0.037
φ and ω scans	θ_max_ = 35.0°, θ_min_ = 1.8°
Absorption correction: multi-scan (SADABS; Bruker, 2005)	*h* = −10→18
*T*_min_ = 0.192, *T*_max_ = 0.688	*k* = −15→15
15460 measured reflections	*l* = −13→13

### Refinement

**Table:**

Refinement on *F*^2^	Primary atom site location: structure-invariant direct methods
Least-squares matrix: full	Secondary atom site location: difference Fourier map
*R*[*F*^2^ > 2σ(*F*^2^)] = 0.037	Hydrogen site location: inferred from neighbouring sites
*wR*(*F*^2^) = 0.092	All H-atom parameters refined
*S* = 1.02	*w* = 1/[σ^2^(*F*_o_^2^) + (0.0376*P*)^2^ + 0.4289*P*] where *P* = (*F*_o_^2^ + 2*F*_c_^2^)/3
3843 reflections	(Δ/σ)_max_ = 0.001
136 parameters	Δρ_max_ = 0.68 e Å^−^^3^
0 restraints	Δρ_min_ = −0.59 e Å^−^^3^

### Special details

**Table:**

Experimental. The low-temperature data was collected with the Oxford Cyrosystem Cobra low-temperature attachment.
Geometry. All esds (except the esd in the dihedral angle between two l.s. planes) are estimated using the full covariance matrix. The cell esds are taken into account individually in the estimation of esds in distances, angles and torsion angles; correlations between esds in cell parameters are only used when they are defined by crystal symmetry. An approximate (isotropic) treatment of cell esds is used for estimating esds involving l.s. planes.
Refinement. Refinement of F^2^ against ALL reflections. The weighted R-factor wR and goodness of fit S are based on F^2^, conventional R-factors R are based on F, with F set to zero for negative F^2^. The threshold expression of F^2^ > 2sigma(F^2^) is used only for calculating R-factors(gt) etc. and is not relevant to the choice of reflections for refinement. R-factors based on F^2^ are statistically about twice as large as those based on F, and R- factors based on ALL data will be even larger.

### Fractional atomic coordinates and isotropic or equivalent isotropic displacement parameters (Å^2^)

**Table:**

	*x*	*y*	*z*	*U*_iso_*/*U*_eq_	
Br1	0.863352 (18)	0.42295 (2)	0.45375 (3)	0.03180 (8)	
N1	0.26074 (15)	0.39137 (18)	0.1162 (2)	0.0234 (3)	
C1	0.50731 (18)	0.31392 (19)	0.4478 (2)	0.0208 (4)	
C2	0.63137 (18)	0.3255 (2)	0.4915 (3)	0.0240 (4)	
C3	0.69332 (18)	0.40815 (19)	0.3962 (2)	0.0222 (4)	
C4	0.63436 (18)	0.4810 (2)	0.2596 (2)	0.0216 (4)	
C5	0.50998 (17)	0.4680 (2)	0.2182 (2)	0.0200 (3)	
C6	0.44549 (16)	0.38295 (19)	0.3101 (2)	0.0184 (3)	
C7	0.31505 (17)	0.3638 (2)	0.2606 (2)	0.0204 (3)	
C8	0.13050 (18)	0.3707 (2)	0.0806 (3)	0.0261 (4)	
C9	0.06720 (18)	0.5093 (2)	0.0273 (3)	0.0245 (4)	
H1	0.462 (2)	0.260 (3)	0.514 (3)	0.028 (6)*	
H2	0.679 (2)	0.273 (3)	0.586 (3)	0.028 (6)*	
H4	0.678 (2)	0.540 (3)	0.209 (3)	0.032 (7)*	
H5	0.470 (2)	0.515 (3)	0.130 (3)	0.024 (6)*	
H7	0.276 (2)	0.330 (2)	0.341 (3)	0.018 (5)*	
H8A	0.113 (2)	0.300 (3)	−0.016 (3)	0.023 (6)*	
H8B	0.1034 (19)	0.336 (2)	0.172 (3)	0.016 (5)*	
H9A	0.111 (3)	0.550 (3)	−0.053 (4)	0.034 (7)*	
H9B	0.092 (3)	0.576 (3)	0.111 (3)	0.033 (7)*	

### Atomic displacement parameters (Å^2^)

**Table:**

	*U*^11^	*U*^22^	*U*^33^	*U*^12^	*U*^13^	*U*^23^
Br1	0.01994 (10)	0.03249 (12)	0.04155 (14)	−0.00088 (8)	0.00144 (8)	−0.00353 (9)
N1	0.0197 (7)	0.0250 (8)	0.0255 (8)	0.0015 (6)	0.0042 (6)	0.0006 (6)
C1	0.0261 (9)	0.0185 (8)	0.0185 (8)	−0.0004 (7)	0.0055 (7)	0.0000 (7)
C2	0.0273 (9)	0.0214 (8)	0.0220 (9)	0.0017 (7)	0.0012 (7)	−0.0003 (7)
C3	0.0221 (8)	0.0208 (8)	0.0237 (9)	0.0000 (7)	0.0041 (7)	−0.0045 (7)
C4	0.0248 (9)	0.0185 (8)	0.0230 (9)	−0.0022 (7)	0.0088 (7)	−0.0014 (7)
C5	0.0239 (9)	0.0175 (7)	0.0194 (9)	0.0003 (7)	0.0060 (7)	0.0009 (7)
C6	0.0204 (8)	0.0156 (7)	0.0196 (8)	0.0008 (6)	0.0048 (6)	−0.0013 (6)
C7	0.0213 (8)	0.0190 (8)	0.0224 (9)	−0.0001 (7)	0.0080 (7)	0.0014 (7)
C8	0.0201 (9)	0.0286 (10)	0.0301 (11)	−0.0004 (7)	0.0055 (8)	0.0020 (8)
C9	0.0200 (8)	0.0248 (9)	0.0285 (10)	−0.0005 (7)	0.0034 (7)	0.0018 (8)

### Geometric parameters (Å, °)

**Table:**

Br1—C3	1.896 (2)	C5—C6	1.398 (3)
N1—C7	1.270 (2)	C5—H5	0.91 (2)
N1—C8	1.458 (3)	C6—C7	1.466 (3)
C1—C2	1.385 (3)	C7—H7	0.92 (2)
C1—C6	1.391 (3)	C8—C9	1.527 (3)
C1—H1	0.95 (3)	C8—H8A	1.04 (2)
C2—C3	1.385 (3)	C8—H8B	0.93 (2)
C2—H2	1.00 (3)	C9—C9^i^	1.512 (4)
C3—C4	1.391 (3)	C9—H9A	0.97 (3)
C4—C5	1.388 (3)	C9—H9B	0.94 (3)
C4—H4	0.90 (3)		
			
C7—N1—C8	117.69 (18)	C1—C6—C7	120.26 (18)
C2—C1—C6	120.93 (19)	C5—C6—C7	120.70 (17)
C2—C1—H1	120.3 (15)	N1—C7—C6	122.12 (18)
C6—C1—H1	118.7 (15)	N1—C7—H7	123.0 (14)
C3—C2—C1	118.83 (18)	C6—C7—H7	114.8 (14)
C3—C2—H2	118.2 (15)	N1—C8—C9	110.13 (18)
C1—C2—H2	122.9 (15)	N1—C8—H8A	107.3 (13)
C2—C3—C4	121.89 (18)	C9—C8—H8A	108.9 (13)
C2—C3—Br1	119.12 (14)	N1—C8—H8B	110.2 (13)
C4—C3—Br1	118.99 (15)	C9—C8—H8B	109.9 (14)
C5—C4—C3	118.31 (19)	H8A—C8—H8B	110.3 (19)
C5—C4—H4	123.6 (17)	C9^i^—C9—C8	112.2 (2)
C3—C4—H4	117.8 (17)	C9^i^—C9—H9A	116.2 (16)
C4—C5—C6	120.99 (18)	C8—C9—H9A	106.1 (16)
C4—C5—H5	119.6 (16)	C9^i^—C9—H9B	116.7 (18)
C6—C5—H5	119.4 (16)	C8—C9—H9B	107.8 (16)
C1—C6—C5	119.02 (17)	H9A—C9—H9B	96 (2)

Symmetry codes: (i) −*x*, −*y*+1, −*z*.
